# Lymphocyte Involvement in the Pathology of Pulmonary Arterial Hypertension

**DOI:** 10.3390/ijms252413455

**Published:** 2024-12-16

**Authors:** Michał Tomaszewski, Agnieszka Styczeń, Martyna Krysa, Adam Michalski, Izabela Morawska-Michalska, Anna Hymos, Joanna Wawer, Agnieszka Rolińska, Mansur Rahnama, Tomasz Urbanowicz, Ewelina Grywalska

**Affiliations:** 1 Department of Cardiology, Medical University of Lublin, Jaczewskiego 8 Street, 20-954 Lublin, Poland; michal.tomaszewski@umlub.pl (M.T.);; 2 Department of Clinical Immunology, Medical University of Lublin, Chodźki 4a Street, 20-093 Lublin, Poland; 3 Department of Experimental Immunology, Medical University of Lublin, Chodźki 4a Street, 20-093 Lublin, Poland; 4 Department of Applied Psychology, Medical University of Lublin, Chodźki 7 Street, 20-093 Lublin, Poland; 5 Department of Dental Surgery, Medical University of Lublin, Chodźki 6 Street, 20-093 Lublin, Poland; 6 Cardiac Surgery and Transplantology Department, Poznań University of Medical Sciences, Fredry 10 Street, 61-107 Poznań, Poland

**Keywords:** pulmonary arterial hypertension, lymphocytes, T-cells, B-cells, immune response

## Abstract

Pulmonary arterial hypertension (PAH) is a disease characterized by increased pulmonary vascular resistance and right heart failure, with emerging evidence suggesting a key role for immune dysregulation in its pathogenesis. This study aimed to assess the involvement of lymphocytes, particularly regulatory T cells (Tregs), and the expression of immune checkpoint molecules PD-1 and PD-L1 on peripheral blood subpopulations in patients diagnosed with PAH. The study involved 25 patients; peripheral blood mononuclear cells were isolated and subsequently analyzed using flow cytometry to quantify the Treg cell percentage and evaluate PD-1 and PD-L1 expression across the T and B cells. We observed a significantly higher percentage of Tregs in idiopathic PAH (iPAH) patients compared to healthy controls and those with congenital heart disease-associated PAH (CHD-PAH), connective tissue disease-associated PAH (CTD-PAH), and chronic thromboembolic pulmonary hypertension (CTEPH). An overexpression of PD-1 and PD-L1 was found on CD4+ and CD8+ lymphocytes in all PAH groups, particularly in iPAH and CHD-PAH patients. These findings align with previous research highlighting Treg dysfunction and PD-1/PD-L1 overexpression as contributors to PAH pathogenesis. Our results suggest that targeting immune checkpoints and modulating Treg function could represent novel therapeutic strategies for PAH. Future research should focus on validating these biomarkers in larger, independent cohorts and exploring their clinical utility in diagnosing and managing PAH.

## 1. Introduction

Pulmonary hypertension (PAH) is a heterogeneous group of diseases associated with increased pulmonary pressure, characterized by a progressive rise in the pulmonary vascular load with remodeling of the pulmonary vasculature. Consequently, it can lead to right ventricular failure and subsequent death [1]. Hemodynamically, the definition of pulmonary hypertension is a mean pulmonary arterial pressure of higher than 20 mmHg at rest during right heart catheterization [2]. Clinical presentation is often non-specific and includes symptoms such as fatigue, chest pain, and fluid retention. An increased pulmonic sound or a tricuspid regurgitation murmur may be heard during physical examination, along with elevated jugular venous pressure or pedal edema [1]. According to the World Health Organization (WHO), pulmonary hypertension can be classified into five clinical groups based on pathophysiology, clinical presentation, hemodynamic characteristics, and management strategies [3]. The prevalence of PAH seems to be greatly underestimated, as it is suspected that approximately 1% of the entire population and approximately 10% of people over 65 years of age suffer from this condition [4]. The leading cause of pulmonary hypertension is left heart disease (LHD) followed by lung diseases, mostly chronic pulmonary disease (COPD) and interstitial lung diseases. Congenital heart diseases (CHD), including valvular and left-sided heart diseases, along with infections such as HIV, can also cause PAH [3,5,6]. The etiopathogenesis is complex and not yet fully understood, especially because of the various possible causes, but there are some common features. One of them is the infiltration of inflammatory cells, which may play a role in originating and/or the propagation of vascular remodeling. There is also the hypothesis of the “bystander phenomenon” where the infiltration of immune cells is a response to ongoing vascular remodeling [7,8].

Regulatory T lymphocytes are responsible for averting autoimmune reactions, as well, as they play a crucial role in maintaining immune homeostasis by limiting vascular injury and enhancing the regenerative properties of different tissues [9,10,11]. The immune balance sustained by regulatory T cells is achieved mostly by the cytokines they secrete with strong anti-inflammatory properties—interleukin-10 (Il-10) and TGF-beta1. They slow down the multiplication and differentiation of other T lymphocytes through the expression of granzyme B and the surface molecule CD73 and weaken the activation of effector T lymphocytes through contact with antigen-presenting cells [12,13]. Recent clinical studies highlight the role of regulatory T lymphocyte dysregulation in the occurrence, progression, and worsening of PAH. These abnormalities contribute to the inability to control inflammation linked to vascular injury from factors like pathogens, shear stress, and ischemia [9,14,15].

With PD-1 being one of the most significant receptors, immune checkpoints play a critical role in their immune-regulatory functions and as key targets for many successful therapies. PD-1, TCR, and CD28 are surface molecules found on T cells. PD-1 is an inhibitory receptor that dampens immune responses, TCR is involved in antigen recognition, and CD28 provides co-stimulatory signals necessary for T cell activation. PD-1 is expressed on both T and B cells following activation, and its ligands, PD-L1 or PD-L2, bind to PD-1, transmitting inhibitory signals that reduce immune responses [16]. PD-L1 is widely expressed in various cells in the human body, including immune cells such as antigen-presenting cells (APCs), T lymphocytes, and B lymphocytes [17]. On T cells, PD-1 suppresses activation signals mediated by the TCR and co-stimulatory receptor CD28, whereas on B cells, PD-1 expression helps modulate the immune response by regulating T cell activity [17,18]. The overexpression of PD-1 and PD-L1 might be linked to chronic lymphocyte activation or exhaustion, leading to functional impairments. Limited literature suggests the involvement of the PD-1/PD-L1 axis in the pathogenesis of PAH, possibly through its impact on regulatory T lymphocytes.

Due to the not fully understood etiology of pulmonary hypertension, with the suspected involvement of immune cells, particularly regulatory T cells, we aimed in our study to assess the percentage of Tregs and the expression of PD-1 and PD-L1 on peripheral blood subpopulations in specific clinical groups of patients with PAH.

## 2. Results

**Figure 1:**
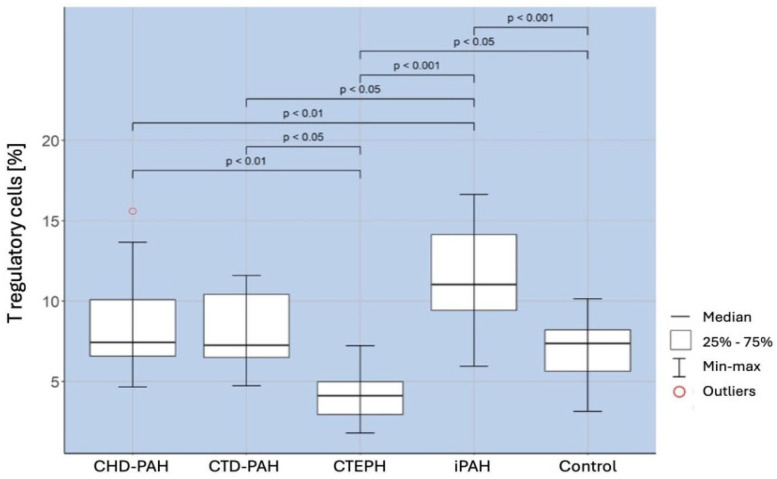
Percentage of regulatory T cells in patients of selected PAH groups and in control groups.

A comparison of the percentage of regulatory T cells in selected types of PAH and the control group showed the existence of a significantly higher percentage of these cells in patients with iPAH than in the control group (*p* < 0.001). There was a significantly lower percentage of Treg in patients with CTEPH (*p* < 0.001), CTD-PAH (*p* < 0,05), and CHD-PAH (*p* < 0.01) compared to iPAH. The obtained correlations are shown in Figure 1.

The percentage of lymphocytes with an expression of immunoregulatory molecules was evaluated. An analysis of the percentage of T (CD4+ and CD8+) and B (CD19+) lymphocytes expressing PD-1/PD-L1 molecules was performed. The results are shown in Table 1.

**Figure 2:**
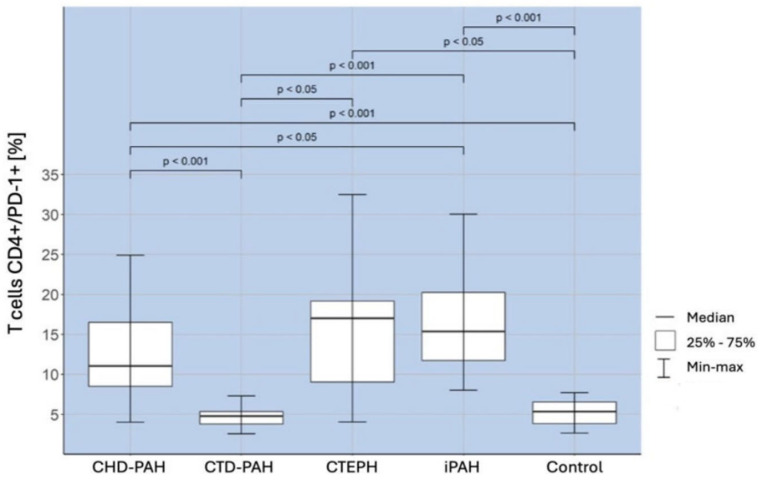
Percentage of CD4+/PD-1+ T lymphocytes in CHD-PAH, CTD-PAH, CTEPH, and iPAH patients and controls.

A comparison of the percentages of CD4+/PD-1+ T lymphocytes in the selected types of PAH and the control group revealed the existence of a significantly higher percentage of these lymphocytes in both the iPAH (*p* < 0.001) and CHD-PAH (*p* < 0.001) groups than in the control and CTD-PAH groups. The obtained correlations are shown in Figure 2.

**Figure 3:**
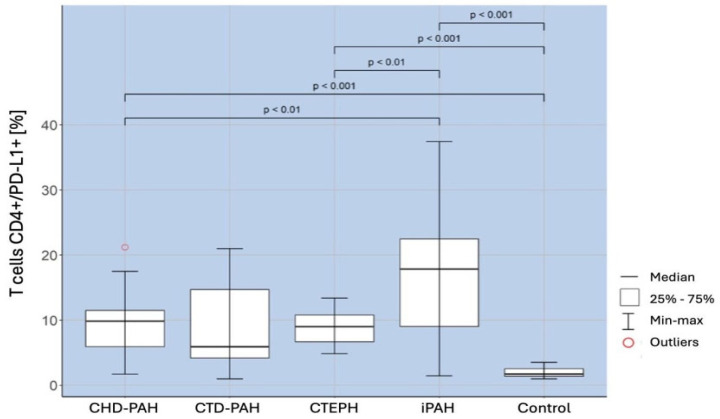
Percentage of CD4+/PD-L1+ T lymphocytes in CHD-PAH, CTD-PAH, CTEPH, and iPAH patients and controls.

A comparison of the percentage of CD4+/PD-L1+ T lymphocytes in selected types of PAH and the control group showed the existence of a significantly higher percentage of these lymphocytes in the iPAH group compared to the control group (*p* < 0.001), the CTEPH group (*p* < 0.01), and the CHD-PAH group (*p* < 0.01). The percentage of CD4+/PD-L1+ T cells was also significantly higher in the CHD-PAH group (*p* < 0.001) and CTEPH group (*p* < 0.001) than in the control group. The obtained correlations are shown in Figure 3.

**Figure 4:**
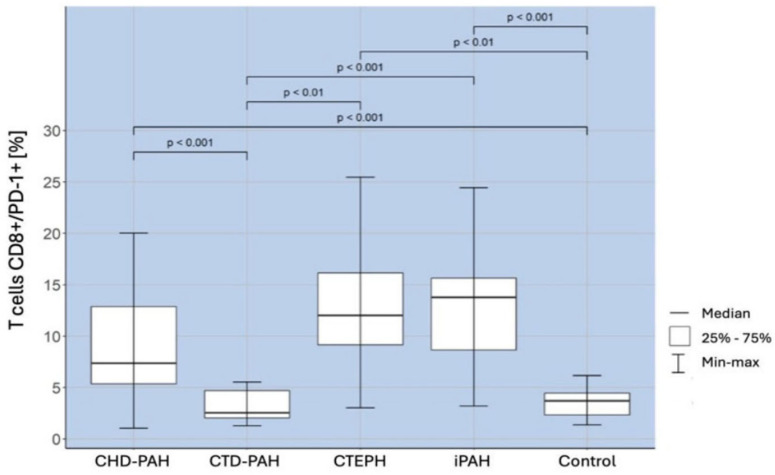
Percentage of CD8+/PD-1+ T lymphocytes in patients with CHD-PAH, CTD-PAH, CTEPH, and iPAH and controls.

Patients in the iPAH group had a significantly higher percentage of T cells CD8+/PD-1+ than the controls (*p* < 0.001) and CTD-PAH patients (*p* < 0.001). Similarly, the percentage was higher in patients with CTEPH than in the controls (*p* < 0.01) and in patients with CTD-PAH (*p* < 0.01). The percentage of CD8+/PD-1+ T cells was also significantly higher in patients with CHD-PAH than in patients with CTD-PAH (*p* < 0.001) and in control subjects (*p* < 0.001). The obtained correlations are shown in Figure 4.

**Figure 5:**
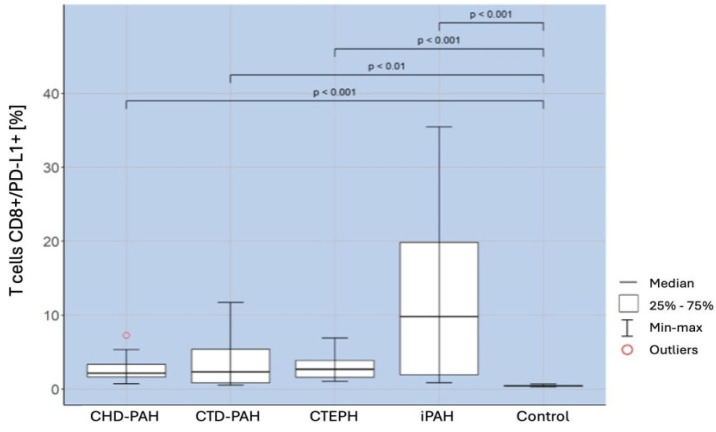
Percentage of CD8+PD-L1+ T cells in CHD-PAH, CTD- PAH, CTEPH, and iPAH patients and controls.

Compared to the control group, patients of all PAH types studied had a significantly higher percentage of CD8+PD-L1+ T cells (*p* < 0.001). The relationships are shown in Figure 5.

## 3. Discussion

**Figure 6:**
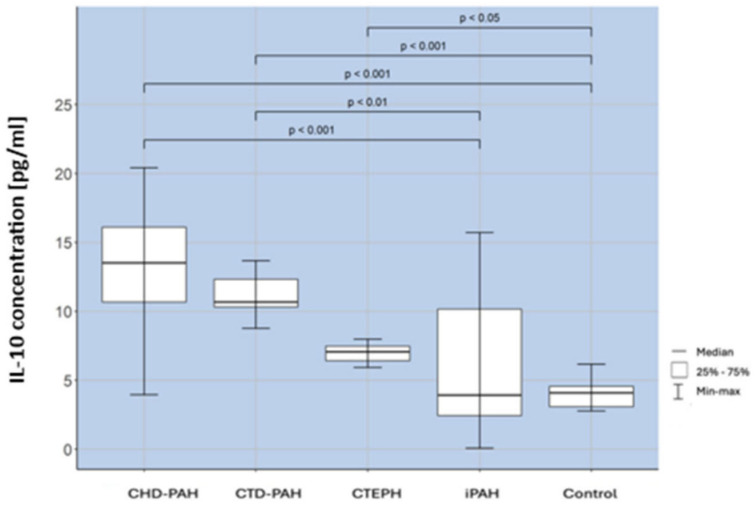
Il-10 concentration assessment in CHD-PAH, CTD- PAH, CTEPH, and iPAH patients and controls.

In our study, we observed a significantly higher percentage of Tregs in patients with iPAH compared to healthy controls. Additionally, differences in Treg levels were noted between various groups of patients with pulmonary hypertension (PH). Patients with iPAH had a significantly higher percentage of Tregs than those with congenital heart disease-associated PAH (CHD-PAH), connective tissue disease-associated PAH (CTD-PAH), and chronic thromboembolic pulmonary hypertension (CTEPH). Our findings align with those of previous studies [19,20]. The increase in Treg levels in iPAH may reflect a compensatory mechanism, though the concurrent inflammation and reduced IL-10 levels, as presented in Figure 6, suggest an ineffective anti-inflammatory response. Regulatory T lymphocytes (Tregs) play a protective role in the pathogenesis of pulmonary arterial hypertension (PAH) [12]. Tregs regulate inflammation through multiple mechanisms, including the release of immunosuppressive cytokines like IL-10 and transforming growth factor-beta (TGF-β), inhibition of dendritic cell function, and disruption of effector cell metabolism—which refers to the metabolic processes that support the function of immune effector cells, such as T cells, B cells, and macrophages, including energy production pathways like glycolysis, oxidative phosphorylation, and fatty acid oxidation, which are crucial for their activation, proliferation, and function [14]. By maintaining a balance in lymphocyte activation, Tregs help prevent harmful immune responses.

Recent studies suggest that Tregs primarily influence the components of the innate immune system, affecting the activation and function of monocytes, macrophages, natural killer (NK) cells, and dendritic cells, rather than regulating the acquired immune response [14]. Tregs have also been shown to suppress the production of pro-inflammatory cytokines such as IL-6 and TNF-α, both of which are linked to worse outcomes in PAH. Elevated IL-6 correlates strongly with mortality in PAH patients, and TNF-α overexpression is associated with poorer prognosis [21,22]. In idiopathic PAH (iPAH), increased levels of circulating Tregs have been previously reported [12,23,24]. The increased percentage of Tregs in iPAH has led researchers to investigate a potential dysregulation in the lymphocyte immune response. Their findings suggest that leptins, which are elevated during acute infections or inflammation, may impair Treg function in iPAH [19,20,25]. Consequently, targeting functional Tregs to reduce inflammatory factor production has emerged as a possible therapeutic approach for PAH patients [10].

Animal models of pulmonary hypertension associated with Treg deficiency demonstrate that the absence of Tregs initiates destructive immunity, driven by macrophage activity. This leads to progressive endothelial damage and vascular remodeling [26,27]. Regulatory T cells are thought to help control macrophage influx and maintain endothelial barrier function, suggesting they may play a role in PAH development [28,29]. While previous studies have reported the dysregulation of regulatory T cells in PAH, our study uniquely focuses on the overexpression of the PD-1/PD-L1 on different lymphocyte subsets (CD4+, CD8+, and CD19+ cells) across multiple types of PAH. This study provides new insights into how immune checkpoint molecules are differentially expressed among PAH subtypes, potentially influencing disease progression and therapeutic responses [9,14,15].

Treg abnormalities contribute to a failure to control inflammation associated with vascular injury from various causes, such as pathogen exposure, shear stress, or ischemia. This dysregulation leads to a prolonged and complex process of vascular wound healing [14,26,29,30,31,32]. Animal studies also indicate that the likelihood of developing severe PAH differs by gender, with females being more susceptible due to the presence of lower Treg levels [33].

In our study, we demonstrated the overexpression of the PD-1 receptor and its ligand on CD4+ and CD8+ T lymphocytes in patients with various types of PAH. In our study, the PD-1 receptor and PD-L1 ligand overexpression on CD4+ lymphocytes was particularly pronounced in patients with iPAH and CHD-PAH, while on CD8+ lymphocytes, it was observed across all patient groups. This is an interesting finding in relation to the disturbances in the number and function of regulatory T lymphocytes in iPAH, strongly suggesting a potential link between these observations. Little is reported about this topic in the literature, as only a few studies have been conducted. It has been shown that PD-1 receptor overexpression, associated with Epstein–Barr virus (EBV) infection, occurs on immune cells in patients with iPAH [34]. One study demonstrated that an increased PD-L1 receptor expression was linked to pyroptosis, pulmonary vascular fibrosis, and potential disease progression [35]. Additionally, cases of PAH development have been reported following the use of anti-PD-1 in cancer immunotherapy [36,37,38]. An overexpression of PD-1 and PD-L1 induced by EBV infection has been observed in various malignancies, including Hodgkin lymphoma and gastric cancers [39,40]. Our previous study [34] found that PAH patients had increased levels of PD-1 and PD-L1 on CD4+ and CD8+ T cells, as well as on B cells. Moreover, we found that the expression of PD-1 on CD4+ T cells correlated with EBV load. Since the PD-1 interaction with its ligand can inhibit lymphocyte activation, proliferation, and expansion, it limits tissue damage and uncontrolled inflammation. Blocking the PD-1/PD-L1 axis in animal models removed the protective function of regulatory T lymphocytes, suggesting that this may increase the risk of developing PAH through the loss of Treg’s protective role [33].

### Study Limitations

This study, despite several limitations, provides a very solid foundation for further investigations into immune dysregulation in PAH. Firstly, the absence of an independent validation cohort limits the robustness and generalizability of our findings. Even though our discovery cohort provided valuable insights, external validation in a larger, multicenter study is necessary to confirm these results. Secondly, the small sample size reduces the statistical power of subgroup analyses and may limit the detection of less pronounced effects. Our study focused on cross-sectional data, which precludes conclusions regarding causality. Lastly, this observational design focuses only on correlations between immune dysregulation and PAH. Furthermore, it does not address the underlying mechanisms. These limitations emphasize the need for further research to explore therapeutic interventions targeting Tregs and PD-1/PD-L1 signaling pathways in PAH.

## 4. Materials and Methods

### 4.1. Patient and Control Group Characteristics

The study involved 70 patients (50 women and 20 men) with a mean age of 57.74 ± 17.17 years (median: 60 years, range: 23–81 years). All patients were treated at the Department of Cardiology at the Medical University of Lublin or the Cardiology Department of the Regional Specialized Hospital in Lublin. Patients diagnosed with PAH were enrolled in this study based on the 2015 European Society of Cardiology (ESC) and European Respiratory Society (ERS) guidelines. Diagnosis was confirmed through right heart catheterization, with a mean pulmonary arterial pressure (mPAP) of ≥25 mmHg at rest, as recommended by the ESC/ERS guidelines [41].

To ensure precise classification into subtypes, patients were categorized into iPAH, CHD-PAH, CTD-PAH, and CTEPH according to WHO classifications. This classification was further corroborated through detailed clinical history, imaging studies, and hemodynamic assessments.

The largest subgroup included 26 patients (19 women and seven men) with PAH related to congenital heart disease (CHD). Other subgroups were idiopathic PAH (iPAH) with 25 patients (15 women and 10 men), chronic thromboembolic pulmonary hypertension (CTEPH) with 10 patients (seven women and three men), and PAH associated with connective tissue disease (CTD) with nine patients (nine women). The World Health Organization (WHO) classification was used to assess heart failure in patients with PAH.

Eligibility criteria included the absence of allergies, infections, immunological disorders, or blood transfusions in the two months prior to the study. All patients underwent a 6-min walk test, measured their B-type natriuretic peptide (BNP) levels, and received a complete blood count. Baseline laboratory tests were conducted at the Alab laboratory of the Independent Public Clinical Hospital in Lublin. Cardiac echocardiography (ECHO) was performed using a Phillips iE33 apparatus, and cardiac catheterization was conducted according to the Polish Society of Cardiology standards [42]. For data analysis, the most recent catheterization result was used. Hemodynamic tests followed ESC guidelines and the national PAH treatment program, which mandates testing at least every 24 months or once in a lifetime for patients with Eisenmenger syndrome.

The control group consisted of 20 individuals with no history of cardiovascular disease, immune modulator treatment, autoimmune diseases, infections, allergies, or recent blood transfusions. To minimize the potential confounding factors, we rigorously applied exclusion criteria to ensure that participants in the control group were free from any conditions that could influence immune parameters.

To address the limitations of cohort size and validation, we conducted an internal validation using statistical resampling techniques. While these methods enhance the reliability of our findings within the discovery cohort, they cannot replace the need for external validation. 

The study protocol received approval from the Bioethics Committee of the Medical University of Lublin (KE-0254/309/2016).

### 4.2. Material

After obtaining written consent, 10 mL of peripheral blood was collected from both the patient and control groups into EDTA-containing tubes using an aspiration-vacuum system (Sarstedt, Nümbrecht, Germany). Immediately following blood collection, lymphocyte immunophenotyping was performed. Peripheral blood mononuclear cells (PBMC’s) were isolated in a density gradient using Gradisol L (Aqua Med, Łódź, Poland), and serum was obtained for future experiments.

### 4.3. Immunophenotyping of Circulating Lymphocytes

The proportion of main lymphocyte subpopulations and expression of PD-1 and PD-1L were determined using flow cytometry. According to the standard protocol, 50 μL of whole blood was stained with 5 μl of each of the anti-human fluorescent conjugated antibodies: CD45 FITC/CD14 PE simultest, CD3 FITC/CD4 PE/CD8 PE-Cy5 simultest, CD3 PE, CD4 FITC, CD8 FITC, CD19 FITC, CD25 PE-Cy5, PD-1 PE, and PD-1L PE (Becton Dickinson, Franklin Lakes, NJ, USA). After 20 min of incubation in the dark at room temperature and washing twice in PBS, labeled cells were analyzed in a FACSCalibur flow cytometer (BD Biosciences, Franklin Lakes, NJ, USA) using CellQuest Pro Software ver. 5.1 (Becton Dickinson, Franklin Lakes, NJ, USA). We have analyzed at least 20,000 cells counted from the lymphocyte gate (gating strategy). Additionally, the number of Treg cells in the CD4+ T lymphocyte subpopulation was determined using the Human Treg Flow Kit (FOXP3 Alexa Fluor 488/CD4 PE-Cy5/CD25 PE), according to the manufacturer’s instructions (Biolegend, San Diego, CA, USA). Cytometric analysis results were presented as the percentage of cells positively stained with the respective monoclonal antibodies.

### 4.4. Statistical Analysis of the Results

Descriptive statistics for continuous variables were presented as minimum and maximum values, median, arithmetic mean, and standard deviation (SD). Comparisons between groups were performed using analysis of variance (ANOVA) with either Duncan’s or Games-Howell post-hoc tests, or the Kruskal–Wallis test with Dunn’s post-hoc test, depending on the data distribution. To compare the means of independent variables, the Student’s *t*-test for independent samples or the Mann–Whitney test was used, depending on whether the criteria for normality of distributions and equality of variances were met. Pearson’s linear correlation coefficient (r) was employed to estimate the correlation between all pairs of variables in each study group. The statistical analyses were conducted using MATLAB 2018b and RStudio (Version 1.1.4.5.6, R package Version 3.5.1). A *p*-value of less than 0.05 was considered statistically significant.

## 5. Conclusions

Our study sheds light on understanding the involvement of T regulatory cells and the potential involvement of the PD-1/PD-L1 axis in the PAH immunoetiopathogenesis. Further studies on a larger group of patients emphasizing specific subpopulations of T and B lymphocytes in different types of PAH are needed. This would enable a more precise determination of the relationship between these cells and different clinical groups of PAH patients.

Our findings underscore the potential role of regulatory T cells and the PD-1/PD-L1 axis in the immunopathogenesis of PAH. While these results are promising, they should be interpreted cautiously due to the limitations of the study design, particularly the absence of an external validation cohort and the small sample size. Future research should focus on validating these biomarkers in larger, independent cohorts and exploring their clinical utility in diagnosing and managing PAH.

## Figures and Tables

**Table 1:** Assessment of the percentage of lymphocytes and the expression of PD-1 and PD-L1.

Parameter	Group	Mean	SD
T CD4+/PD-1+ lymphocytes [%]	CHD-PAH	12.21	5.29
	CTD-PAH	5.04	1.83
	CTEPH	15.53	8.34
	iPAH	17.96	7.35
	Control group	5.35	1.54
T CD4+PD-L1+ lymphocytes [%]	CHD-PAH	9.84	5.25
	CTD-PAH	8.92	6.66
	CTEPH	8.93	2.77
T CD4+PD-L1+ lymphocytes [%]	iPAH	17.46	9.94
	Control group	1.86	0.7
T CD8+/PD-1+ lymphocytes [%]	CHD-PAH	8.85	5
	CTD-PAH	3.18	1.58
	CTEPH	13.25	6.75
	iPAH	12.89	5.38
	Control group	3.6	1.46
T CD8+PD-L1+ lymphocytes [%]	CHD-PAH	3.92	4.08
	CTD-PAH	3.47	3.64
	CTEPH	2.98	1.84
	iPAH	12.23	10.83
	Control group	0.45	0.11
CD19+/PD-1+ lymphocytes [%]	CHD-PAH	3.17	3.51
	CTD-PAH	1.54	1.17
	CTEPH	6.66	4.18
	iPAH	5.35	4.55
	Control group	1.67	0.84
B CD19+PD-L1+ lymphocytes [%]	CHD-PAH	4.2	3.64
	CTD-PAH	2.39	1.67
	CTEPH	3.19	2
	iPAH	10.59	7.59
	Control group	0.26	0.22

Abbreviations: CHD-PAD—congenital heart disease–pulmonary arterial disease, CTD-PAH—connective tissue disease–pulmonary arterial hypertension, CTEPH—chronic thromboembolic pulmonary hypertension, iPAH—idiopathic pulmonary arterial hypertension, SD—standard deviation.

## Data Availability

Due to privacy and ethical concerns, the data supporting this study’s findings are available on request from the first author (M.T.).
